# Supplementary material for: One-milligram torsional pendulum toward experiments at the quantum-gravity interface
Source: Commun Phys. 2026 Jan 30;9(1):80. doi: 10.1038/s42005-026-02514-w (PMC12960207; doi:10.1038/s42005-026-02514-w)
Supplement: Supplementary file 1 — Supplementary information [file 42005_2026_2514_MOESM1_ESM.pdf]

Supplementary Information
for
One-milligram torsional pendulum toward experiments at the quantum-gravity interface

Contents

Supplementary Notes 1: Experimental Methods **S1**
1.1 Optimal tilt sensing with a split photodiode S1
1.2 Push-beam power control S3
1.3 Yaw-motion feedback control and its noise S3
1.4 Taking under control other pendulum modes S5
1.5 On noise sources S6
1.6 Torque sensitivity S9

Supplementary Notes 2: Theoretical Modeling **S9**
2.1 Quantum coherence length and purity S9
2.1.1 Thermal harmonic oscillator S10
2.2 Damped harmonic oscillator S11
2.2.1 Damping via thermal bath S11
2.2.2 Quantum feedback damping S12
2.2.3 Experimental extraction of coherence length and purity S14
2.3 Figure of merit η for different systems S15
2.3.1 On the adapted form of η S15
2.3.2 Geometry factors for evaluating $|\nabla F|$ S16
2.3.3 Parameters used for η comparisons S18
2.3.4 On the potential future performance S20

Supplementary Note 1: Experimental Methods

1.1 Optimal tilt sensing with a split photodiode

Here, we derive the optimal sensitivity achievable for tilt sensing using a split photodiode, and introduce the tilt sensitivity parameter utilized in the main text.

After reflection of a Gaussian beam incident on a pendulum tilted by an angle θ, the beam will acquire a 2θ tilt with respect to an untilted reference axis. A beam tilt

by angle 2θ is equivalent to introducing a position-dependent phase shift $2\theta k_0 x$ on the beam, where x is the coordinate perpendicular to the propagation axis and $k_0 = 2\pi/\lambda$ is the wave number in terms of the wavelength λ. For small angles, the phase factor can be expressed as $e^{i2\theta k_0 x} \approx 1 + i2\theta k_0 x$, and the reflected beam profile can be described as a sum of zeroth-order and first-order Hermit-Gauss modes, as we will illustrate. In the following, we will make use of the normalized Hermite-Gauss mode functions

$$\begin{aligned} \phi_0(x,z) &= \tfrac{2^{1/4}}{(\pi \mathrm{w}^2(z))^{1/4}} e^{-x^2/\mathrm{w}^2(z)} e^{-ik_0 x^2/2R(z)} e^{i\varphi(z)} \\ \phi_1(x,z) &= \phi_0(x,z) \tfrac{2x}{\mathrm{w}(z)} e^{i\varphi(z)}. \end{aligned} \tag{S1}$$

Here, z is the propagation distance along the reference axis, and $\mathrm{w}(z)$, $R(z)$ and $\varphi(z)$ are the propagation-distance-dependent spot size, wavefront curvature, and Gouy phase, respectively. The Gouy phase covers a range of π radians as z goes from $-\infty$ to ∞.

We will take the pendulum to be located at z_p. Right after the pendulum, the incident beam represented by the wave $\psi(x, z_\mathrm{p}) = \phi_0(x, z_\mathrm{p})$ at the location of the pendulum will evolve into

$$\begin{aligned} \psi'(x,z_\mathrm{p}) &= \phi_0(x,z_\mathrm{p}) e^{ik_0 2\theta x} \\ &\approx \phi_0(x,z_\mathrm{p}) + ie^{-i\varphi(z_\mathrm{p})}\theta k_0 \mathrm{w}(z_\mathrm{p}) \phi_1(x,z_\mathrm{p}) \end{aligned} \tag{S2}$$

indicating that the tilt scatters a small amplitude into the first-order Hermit-Gauss mode which is proportional to the spot size $\mathrm{w}(z_\mathrm{p})$ at the location of the pendulum. To quantify the fundamental sensitivity to a tilt, first note that Eq. S2 is compatible with the physical propagating beam given by the wave

$$\begin{aligned} \Psi(x,z) &= \phi_0(x,z) + ie^{-i\varphi(z_\mathrm{p})}\theta k_0 \mathrm{w}(z_\mathrm{p}) \phi_1(x,z) \\ &= \phi_0(x,z) \left(1 + ie^{i(\varphi(z)-\varphi(z_\mathrm{p}))} x \tfrac{2\theta k_0 \mathrm{w}(z_\mathrm{p})}{\mathrm{w}(z)}\right), \end{aligned} \tag{S3}$$

since $\Psi(x, z_\mathrm{p}) = \psi'(x, z_\mathrm{p})$. A split detector measures the shift in the position of a beam by differencing the integrated intensities (proportional to $|\Psi(x, z)|^2$) in the two halves of the space. We can define a metrologically relevant sensitivity parameter $\mathcal{S}$ magentaas the change of the split detector signal as a function of tilt angle:

$$\begin{aligned} \mathcal{S}(z) &= \frac{d}{d\theta} \left| \int_{-\infty}^{0} |\Psi(x,z)|^2 dx - \int_{0}^{\infty} |\Psi(x,z)|^2 dx \right| \\ &= \tfrac{\sqrt{32\pi}}{\lambda} \mathrm{w}(z_\mathrm{p}) |Re[ie^{i(\varphi(z)-\varphi(z_\mathrm{p}))}]| \end{aligned} \tag{S4}$$

Here, terms proportional to θ^2 have been omitted in evaluating the integral as per the small-angle approximation. $\mathcal{S}$ is related to the mean displacement-to-spot size ratio as $\mathcal{S}(z) = \sqrt{\frac{8}{\pi}} \frac{d}{d\theta} \frac{\langle x \rangle_z}{\mathrm{w}(z)}$, where $\langle x \rangle_z$ is the z-dependent mean displacement. As a remark for physical insight, we note that the quantity $1/(\mathcal{S}\ \theta)^2$ coincides with the number of

photons needed to resolve θ in an experiment that is limited by photon counting noise for tilt measurements—see for example [1].

Given the range of the Gouy phase function $\varphi(z)$, mathematically, there always exists a location $z_{\rm opt}$ where $|Re[ie^{i(\varphi(z)-\varphi(z_{\rm p}))}]| = 1$ that maximizes $\mathcal{S}(z)$ to a value independent of the beam size at $z_{\rm opt}$:

$$\mathcal{S}_{\max} \equiv \mathcal{S}(z_{\rm opt}) = \frac{\sqrt{32\pi}}{\lambda}\,\mathrm{w}(z_{\rm p}) \tag{S5}$$

This is the fundamental upper limit to our tilt sensitivity parameter. In the main text, $\mathrm{w}(z)$ and $\mathrm{w}(z_{\rm p})$ are replaced with w and w_p, respectively, and $\langle x\rangle_z$ is replaced with $\delta(\theta)$ for notational simplicity.

The maximization performed does not readily provide the spot size $\mathrm{w}(z_{\rm opt})$ where $\mathcal{S}$ is maximized. It could happen that the beam is very small at this point and falls into the gap of the split detector. Nevertheless, this can be remedied by additional beam shaping following the tilt, since a set of lenses can be utilized to independently adjust the Gouy phase shift and the beam size as illustrated in Figure 1(e) of the main text.

1.2 Push-beam power control

The push beam power reflecting from the pendulum is incident on a photodiode, and the measured power is stabilized by a 300-kHz-bandwidth analog feedback circuit. The electronically variable set-point of the stabilization determines the push power the pendulum sees. The set-point takes in a sum of three input signals: 1) a precision DC voltage for setting the default operating power, 2) a signal for arbitrary power modulation, and 3) a signal coming from the quadrant photodiode for engineering feedback-based equations of motion. The modulation input of this circuitry was utilized in obtaining the susceptibility curves in Fig. 2(a), where a white noise signal was input to generate a driving torque. In the absence of the second and third inputs, the push beam operates at a midpoint power of 2 mW with near-shot-noise-limited intensity fluctuations at all relevant frequencies.

1.3 Yaw-motion feedback control and its noise

Here, we explain the feedback circuit that controls the yaw motion, and derive the effective susceptibility as well as the feedback noise in the system. The feedback loop is illustrated in Fig. S1. Expressed in the frequency domain, the controlled output of the system is the pendulum angle $\theta(\omega)$. This output is detected with an added angle equivalent imprecision noise $\delta\theta_{\rm imp}(\omega)$, and fed into a loop filter with a transfer function $G(\omega)$ determining how this signal is converted to an *active* feedback torque $\tau_{\rm act}(\omega)$ through the push beam. Additional *passive* torque noises $\delta\tau_{\rm pas}(\omega)$ also act on the system. The applied torques turn into a yaw angle through the natural mechanical susceptibility (torque-to-angle transfer function) $\chi(\omega) = (\omega_0^2 - \omega^2 + i\omega\gamma(\omega))^{-1}/I$, completing the loop. The passive torques consist of the thermal torque noise, the radiation pressure noise of the probe and push beams, and torques induced by vibration noise around the setup. In the current work, intensity fluctuations are near shot-noise

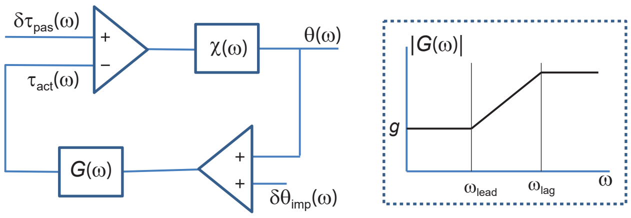

Fig. S1 Schematic of the feedback loop for yaw motion control.

limited, and the resulting total radiation pressure noise is at least three orders of magnitude smaller than thermal noise.

Self-consistency in the loop yields $\theta = \chi(\delta\tau_{\text{pas}} - \tau_{\text{act}})$ with $\tau_{\text{act}} = G(\theta + \delta\theta_{\text{imp}})$. Solving for θ gives

$$\theta(\omega) = \chi_{\text{eff}}(\omega)\delta\tau_{\text{pas}}(\omega) - \chi_{\text{imp}}(\omega)\delta\theta_{\text{imp}}(\omega), \tag{S6}$$

where the effective susceptibility is $\chi_{\text{eff}}(\omega) = \frac{\chi(\omega)}{1+G(\omega)\chi(\omega)}$, and the imprecision noise transfer function is $\chi_{\text{imp}}(\omega) = G(\omega)\chi_{\text{eff}}(\omega)$. The dimensionless loop gain $G(\omega)\chi(\omega)$ needs to satisfy the usual stability criterion ($< 180°$ phase shift at the unity loop gain) for the loop to be stable.

The loop transfer function is chosen as that of a variable-gain lead-lag compensation filter (Fig. S1)

$$G(\omega) = \frac{g}{\chi(0)} \frac{1 + i\omega/\omega_{\text{lead}}}{1 + i\omega/\omega_{\text{lag}}} \approx \frac{g}{\chi(0)}(1 + i\frac{\omega}{\omega_{\text{lead}}}). \tag{S7}$$

Here, ω_{lead} and ω_{lag} are the start and stop frequencies of the linear gain increase, and the filter gain is chosen such that the DC loop gain $G(0)\chi(0) = g$. The approximation in Eq. S7 is valid for $\omega_{\text{lead}} \ll \omega_{\text{lag}}$. In our experiment, $\omega_{\text{lag}} = 2\pi \times 1.5$ kHz and $\omega_{\text{lead}} = \omega_{\text{eff}} Q_{\text{eff}}(1 - \frac{\omega_0^2}{\omega_{\text{eff}}^2})$ has a minimum value of $2\pi \times 7.7$ Hz for the case of $\omega_{\text{eff}} = 2\pi \times 18$ Hz with critical damping $Q_{\text{eff}} = 1/2$, satisfying the approximation well. The unity gain point of the feedback loop needs to come before ω_{lag} for loop stability, but the lag part of the filter is needed to make $G(\omega)$ physical since no filter can have an endless gain increase with frequency. Utilizing the parameters in S7, the effective susceptibility $\chi_{\text{eff}}(\omega)$ is given by

$$\begin{aligned}\chi_{\text{eff}}^{-1}(\omega) &= \chi^{-1}(\omega) + G(\omega) \\ &= I(\omega_{\text{eff}}^2 - \omega^2 + i\omega\gamma_{\text{eff}})\end{aligned} \tag{S8}$$

where $\omega_{\text{eff}} = \omega_0\sqrt{1+g}$ and $\gamma_{\text{eff}} = \frac{\omega_0^2 g}{\omega_{\text{lead}}} + \gamma(\omega)$. Taking into account that $\gamma_{\text{eff}} \gg \gamma(\omega)$ and $\chi(0) = (I\omega_0^2)^{-1}$, Eq. S7 can be rewritten as $G(\omega) = I(\omega_{\text{eff}}^2) - \omega_0^2 + i\omega\gamma_{\text{eff}}$, resulting

into an explicit expression for the imprecision transfer function

$$\chi_{\mathrm{imp}} = \frac{(\omega_{\mathrm{eff}}^2) - \omega_0^2 + i\omega\gamma_{\mathrm{eff}}}{(\omega_{\mathrm{eff}}^2) - \omega^2 + i\omega\gamma_{\mathrm{eff}}} \tag{S9}$$

Note that all expressions that contain γ_{eff} can also be written in terms of $Q_{\mathrm{eff}} = \frac{\omega_{\mathrm{eff}}}{\gamma_{\mathrm{eff}}}$, which is the primary quantity utilized for the experimental analysis in the main text.

Since all torque and measurement imprecision noises are mutually uncorrelated, the oscillator angular PSD can simply be expressed as

$$S_{\theta\theta}(\omega) = |\chi_{\mathrm{eff}}(\omega)|^2 S_{\tau\tau}^{\mathrm{pas}}(\omega) + |\chi_{\mathrm{imp}}(\omega)|^2 S_{\theta\theta}^{\mathrm{imp}}(\omega), \tag{S10}$$

where the total passive torque PSD $S_{\tau\tau}^{\mathrm{pas}}$ is dominated by the suspension thermal noise with a small contribution from vibrations: $S_{\tau\tau}^{\mathrm{pas}}(\omega) \approx S_{\tau\tau}^{\mathrm{th}}(\omega) + S_{\tau\tau}^{\mathrm{vib}}(\omega)$.

In comparison to a simple change in susceptibility, Eq. S10 shows that the feedback control additionally imprints some detection noise on the oscillation angle PSD. This becomes relatively more important only when the feedback loop tries to control the oscillation amplitude at the measurement noise limit. Eq. S10 forms the basis of the spectral motion analysis under active control in the main text.

1.4 Taking under control other pendulum modes

Robust manipulation of the yaw motion requires first taking many other modes of the pendulum under control. In our case, the primary reason for this surfaced when we needed to float the optical table hosting the experiment to reduce ground vibration noise above 5 Hz, such that thermal-noise-limited operation could be achieved. However, floating the table initially rendered the system unworkable due to the large swing-mode motions it induced. Another limitation was the yaw-motion feedback leading to instabilities in the higher-frequency violin modes of the pendulum.

To circumvent these problems, we mounted the vacuum chamber on the optical table from a single side, forming a cantilever-like structure with natural resonance around 65 Hz. We then actuated (pushed on) the chamber from two orthogonal directions with piezo transducers, gaining the capability of jiggling the pendulum suspension point in space. This led to a configuration where all modes other than the yaw mode were strongly affected by the piezos. The yaw motion was near-purely actuated by the push beam — due to the natural decoupling of torsional motion from suspension point motion. Note that the optical lever system and the QPD were mounted on the rigid optical table, measuring the true motion of the pendulum relative to the massive optical table.

Various levels of information were available on the QPD output for each of the pendulum modes, e.g., longitudinal and horizontal swings at 2.27 Hz, roll at 44.48 Hz, pitch at 125.95 Hz, 1st longitudinal and transverse violins at 74.40 Hz and 82.28 Hz, and 2nd longitudinal and transverse violins at 169.59 Hz and 178.80 Hz, etc. Note that for an ideal pendulum, no information is expected on the optical lever about the modes that give rise to motion in the transverse direction. However, a 2-degree tilt (from vertical) of the pendulum surface that resulted from a non-ideal gluing of the

suspension fiber resulted in all transverse modes imprinting some information on the optical lever.

Each spectral line in the QPD signal was appropriately band-passed, phase-shifted and amplified with analog electronics and fed back to the relevant piezo transducer to sufficiently dampen the corresponding mechanical mode. These additional feedback channels were not intended to induce strong cooling effects in all modes. To give an idea of the level of control utilized for these other modes, we first note that the roll, pitch and violin modes all displayed free-running quality factors in the range 3×10^5 to 5×10^5 as estimated from the QPD signal spectra, and the swing modes displayed a quality factor of 1.0×10^6 as characterized from a dedicated ring-down measurement. The utilized feedback signals reduced the effective quality factors of all these modes. During typical operation, the steady state amplitudes were reduced, for example, by a factor of about 70 for the swing modes, about a factor of 10^3 for the roll and the 1st-longitudinal violin modes, and about a factor of 500 for the 1st-transverse violin and the pitch modes. Such operation ensured that potential large swing motions did not saturate the detection system, and that even for the strongest feedback strengths on the torsional mode, the resonances associated with other modes were not rendered unstable due to residual cross couplings of the torsion feedback to any other mode with the wrong sign—which would give rise to amplification. Additional details about this far-from-optimal feedback loop are beyond the scope of this article; however, the discussed challenges are not a show stopper for scaling the current setup to more sensitive versions. They just indicate that the vibration isolation architecture needs to be planned in advance to alleviate limitations due to ambient vibrations.

1.5 On noise sources

Various noise sources can in principle plague optomechanical control experiments at low frequencies. These include detection noise, which can be imprinted inadvertently onto the motion under feedback control (Eq. S10); seismic noise, which can excite the motion beyond that induced by thermal noise; actuation noise, leading to the same; and quantum radiation pressure noise. In the operating parameter regime of this experiment, no noise source other than thermal torque noise and detection noise is relevant, as they are considerably subdominant and are not directly measurable in many cases.

For example, the torsional motion is actuated by the push beam whose intensity fluctuations are stabilized at its shot noise level, corresponding to a white-noise quantum radiation pressure level at least three orders of magnitude smaller than thermal torque noise. The electronic noise in the actuation is of the same order as the radiation pressure noise itself. Furthermore, no observable noise above the thermal noise floor is inadvertently coupling to the yaw motion from the weak piezo actuation that is used to keep under control the modes other than yaw.

The seismic vibration levels are immeasurable in the frequency band of interest under the passive pneumatic vibration isolation of the optical table. As stressed in the main text, one of the main reasons for the interest of utilizing torsional motion is its inherent isolation from environmental vibrations. The suppression of the effects of seismic noise to well below the thermal torque noise level is evidenced in Fig. S2,

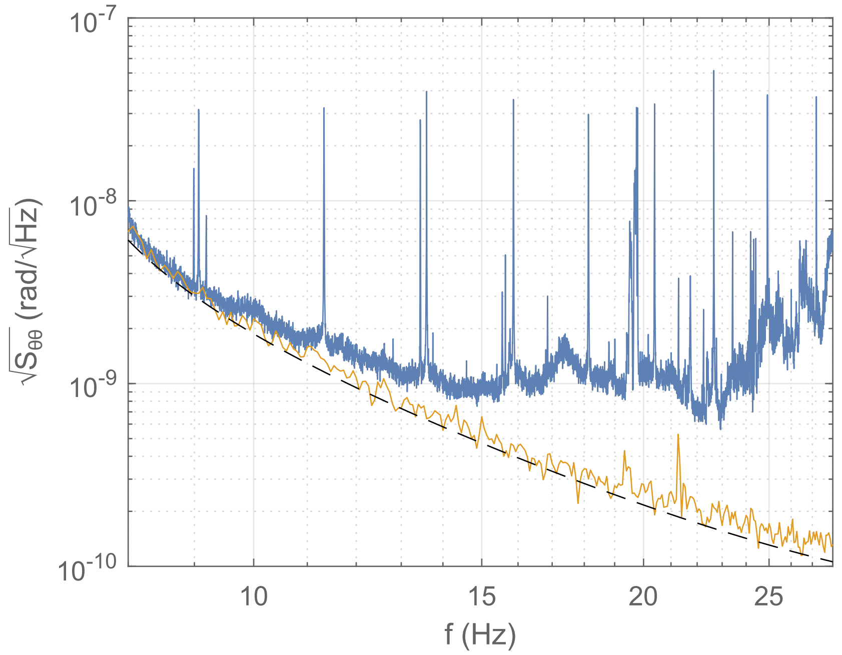

Fig. S2 Vibration noise in the frequency band of interest with (yellow) and without (blue) the optical table floating. With the vibration isolation provided by the floating table, the yaw motion noise spectrum follows the curve (dashed) expected due to the thermal noise floor.

where it is observed that once the optical table is floated, the yaw motion becomes thermal-noise-limited.

For detection noise, here we provide a more extended characterization than given in Fig. 1(c) of the main text, which was sufficient to analyze the main experiment in the relevant frequency band of 8-28 Hz. In particular, here we include lower frequencies. This is relevant as we are observing a clear characteristic structural-damping noise spectrum all the way down to 0.1 Hz, but, in the absence of a characterization at low frequencies, Fig. 1(c) might suggest that the detection noise could even be above the observed thermal noise. The new characterization was carried out in a separate, but very similar optical lever setup under very similar conditions, again with a rigid mirror, to understand the intrinsic noise originating only from the lever itself.

The new and the old detection noise characterizations are shown in Fig. S3. It is important to note that while the main experiments and the new detection noise characterizations were carried out under a well-sealed cardboard enclosure, the old detection noise characterizations were done under a non-ideally sealed enclosure. The abrupt noise increase below 8 Hz in the old characterization originates from air currents on the optical lever path at low frequencies. Once eliminated with the use of the enclosure, the underlying 1/f-type noise—with a strength well below the measured thermal noise spectrum— becomes apparent at low frequencies. This noise originates from the beam-pointing fluctuations of the optical lever beam launched to the setup.

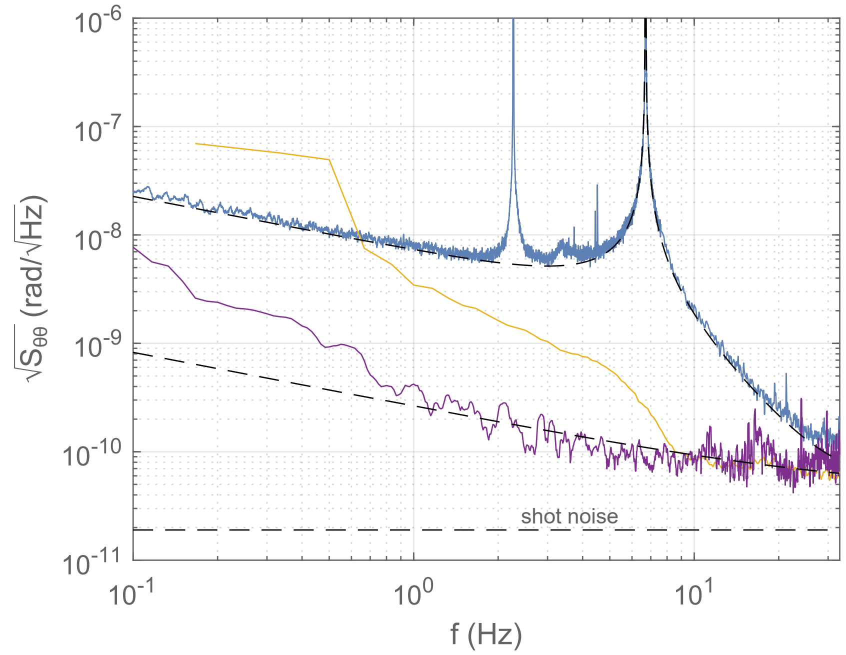

Fig. S3 Additional characterization of detection noise in a similar optical lever setup. The old (yellow) and the new (purple) noise characterizations are shown together with a 1/f curve (dashed line) that fits well to the new noise floor down to 1 Hz. The measured thermal noise spectrum (blue), as well as the expected shot noise level are also shown.

This inference is supported by the following observations: The fluctuations still persist in the absence of an intermediate rigid mirror, i.e., when the beam is launched straight on the QPD, and in this configuration the sum channel of the QPD sensing the total power reads at shot noise levels while the difference channel sensing the beam displacement is still showing the 1/f-type excess noise, whose magnitude scales linearly with the input optical power as expected. Note that for all the data analysis in the main text, the noise characteristics of the original optical lever in the relevant frequency band are utilized— Fig. S3 is only for informational purposes.

We would like to stress that in our parameter range of operation, for feedback-based frequency shifting or cooling, there is no special role played by a shot-noise-limited detection. This would become important only close to the quantum ground state. All that is required then is that the detection noise spectrum lies below the torsional motion spectrum during feedback control at any utilized feedback strength—looking at Fig. 2b we see that this is the case even at critical damping. If the motion can be measured with a certain reliability, it can typically also be controlled at that reliability, irrespective of the source of the detection noise. For example, we could have in principle lowered the power in the optical lever to the point that we were no longer resolving the intrinsic pointing noise in the optical lever, and were then detection shot-noise-limited. No qualitative change would have taken place in our experiments.

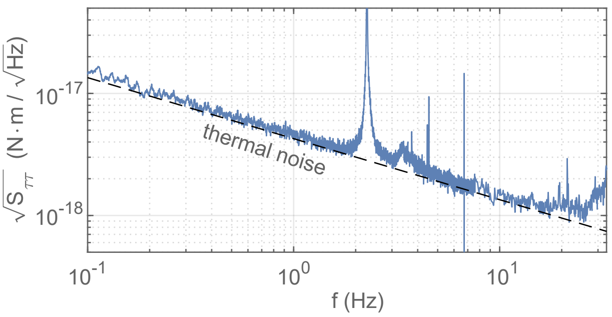

Fig. S4 Torque sensitivity. Free running torque-referred angular noise $S_{\tau\tau} \equiv S_{\theta\theta}/|\chi|^2$, characterizing the torque sensitivity. The loss of sensitivity around 2.2 Hz is due to leakage of the pendulum swing mode signals into the yaw channel.

1.6 Torque sensitivity

In addition to the demonstrated optomechanical control capabilities, the developed pendulum system is very competitive as a sensor. The torque sensitivity is primarily limited by structural thermal noise, and reaches a minimal observed value of 1.2×10^{-18} around 20 Hz (Fig. S4). This corresponds to a broadband angular acceleration sensitivity of 3.3×10^{-6} rad s^{-2} Hz$^{-1/2}$, or, when referred to the tip of the pendulum, to a broadband linear acceleration sensitivity of 3.3×10^{-10} g Hz$^{-1/2}$. This value is on par with state-of-the-art *resonant* acceleration sensitivities achievable with magnetically levitated particles at the 1-milligram level in cryogenic systems [2].

While achieving a factor of 10 beyond state-of-the-art torque sensitivity at the milligram scale [3], this thermal-noise-limited sensitivity allows for a very clear demonstration of the peculiar properties of structural damping for nearly three decades of a frequency range in our setup — 1/f noise in $S_{\tau\tau}(f)$ instead of white noise.

Supplementary Note 2: Theoretical Modeling

2.1 Quantum coherence length and purity

Following pioneering interference experiments with trapped ions, the concept of a coherence length for matter waves was rigorously developed in references [4, 5], in analogy to optical coherence. The main outcome that is useful for our purposes is the definition of the coherence length ξ:

$$\xi^2 = \frac{Tr(\hat{\rho}^2\hat{x}^2) - Tr(\hat{\rho}\hat{x}\hat{\rho}\hat{x})}{Tr(\hat{\rho}^2)}. \tag{S11}$$

Here, $\hat{\rho}$ is the quantum-mechanical density operator and $\hat{x}$ is the position operator of the object. In terms of the density matrix elements $\rho(x, x') = \langle x|\hat{\rho}|x'\rangle$, the above

expression can be written as

$$\xi^2 = \frac{\int_{-\infty}^{\infty}\int_{-\infty}^{\infty} dxdx'\ |\rho(x,x')|^2 \frac{1}{2}(x-x')^2}{\int_{-\infty}^{\infty}\int_{-\infty}^{\infty} dxdx'\ |\rho(x,x')|^2} \tag{S12}$$

Changing variables to $x \to x + \frac{\Delta}{2}$ and $x' \to x - \frac{\Delta}{2}$ to make the dependence on the off-diagonal ($x \neq x'$) elements explicit, and defining the shorthand notation $|g(\Delta)|^2 = \int_{-\infty}^{\infty} dx|\rho(x+\frac{\Delta}{2}, x-\frac{\Delta}{2})|^2$, we reach a compact form of the coherence length equation

$$\xi^2 = \frac{1}{2}\frac{\int_{-\infty}^{\infty} d\Delta\ |g(\Delta)|^2\Delta^2}{\int_{-\infty}^{\infty} d\Delta\ |g(\Delta)|^2}. \tag{S13}$$

Here, $|g(\Delta)|^2$ is a position-averaged version of the off-diagonal elements of the density matrix. Equation S13 extracts the mean range of the off-diagonal density matrix elements, forming a direct relationship to fringe visibility in matter wave interference experiments.

The purity of a quantum state, defined as $\mathcal{P} = Tr[\hat{\rho}^2]$, takes the form

$$\mathcal{P} = \int_{-\infty}^{\infty} d\Delta\ |g(\Delta)|^2. \tag{S14}$$

The purity reaches a maximum value of one, and approaches zero for a completely mixed state.

2.1.1 Thermal harmonic oscillator

In the Sudharsan P representation, a thermal state of a harmonic oscillator can be expressed as an incoherent mixture of coherent states $|\alpha\rangle$ with $\alpha = \alpha_{\mathrm{re}} + i\alpha_{\mathrm{im}}$ the complex amplitude:

$$\hat{\rho} = \int d^2\alpha \frac{1}{\pi n_{\mathrm{th}}} e^{-|\alpha|^2/n_{\mathrm{th}}} |\alpha\rangle\langle\alpha|. \tag{S15}$$

Here n_{th} is the mean thermal excitation quanta, and the integration is over the whole complex plane. Given the projection onto the position basis elements $\langle x|\alpha\rangle = (\frac{1}{2\pi x_{\mathrm{zp}}^2})^{1/4}\exp[-\frac{(x-2x_{\mathrm{zp}}\alpha_{\mathrm{re}})^2}{4x_{\mathrm{zp}}^2} + i\frac{\alpha_{\mathrm{im}}x}{x_{\mathrm{zp}}} - i\alpha_{\mathrm{im}}\alpha_{\mathrm{re}}]$, where x_{zp}^2 is the ground state position variance, the density matrix elements can be expressed as

$$\rho(x+\tfrac{\Delta}{2}, x-\tfrac{\Delta}{2}) = \sqrt{\tfrac{1}{2\pi\sigma_x^2}} e^{-x^2/2\sigma_x^2} e^{-\Delta^2/2\sigma_\Delta^2}, \tag{S16}$$

where $\sigma_x^2 = x_{\mathrm{zp}}^2(2n_{\mathrm{th}}+1)$ and $\sigma_\Delta^2 = 4x_{\mathrm{zp}}^2/(2n_{\mathrm{th}}+1)$. Thus, for the coherence length ξ, the position uncertainty $\Delta x \equiv Tr[\hat{\rho}\hat{x}^2]^{1/2}$ and the purity $\mathcal{P}$, we obtain

$$\begin{aligned} \xi &= \tfrac{1}{2}\sigma_\Delta = x_{\mathrm{zp}}/\sqrt{2n_{\mathrm{th}}+1}, \\ \Delta x &= \sigma_x = x_{\mathrm{zp}}\sqrt{2n_{\mathrm{th}}+1}, \\ \mathcal{P} &= \tfrac{\xi}{\Delta x} = 1/(2n_{\mathrm{th}}+1). \end{aligned} \tag{S17}$$

These expressions indicate that the coherence length shrinks with increasing temperature in contrast to a growing overall position uncertainty — with the product $\xi \cdot \Delta x = x_{\mathrm{zp}}^2$ constant.

2.2 Damped harmonic oscillator

In this section, we will describe the behavior of a damped harmonic oscillator from an open quantum systems point of view, using master equations. We will see how the system equilibrates in a thermal quantum state. We will look into both the case of damping due to coupling to a high-temperature bath and the more complicated case of active feedback damping. For the latter case, we will see deviations from the ideal thermal state result near the oscillator's ground state, as well as when the system approaches the overdamped region. Our main focus will be on extracting coherence lengths.

2.2.1 Damping via thermal bath

A master equation for a harmonic oscillator coupled to a thermal bath was derived in reference [6] in the Markov and Born approximations and further studied in the density matrix formalism, for example, in references [7–9] to understand quantum coherence and interference phenomena in damped systems. The density matrix master equation reads

$$\tfrac{d}{dt}\hat{\rho} = \tfrac{1}{i\hbar}[\hat{H}_0, \hat{\rho}] + \tfrac{1}{i\hbar}\tfrac{\gamma_0}{2}[\hat{x}, \hat{p}\hat{\rho} + \hat{\rho}\hat{p}] - \tfrac{D_{\mathrm{th}}}{\hbar^2}[\hat{x}, [\hat{x}, \hat{\rho}]]. \tag{S18}$$

The first term in the rate of change of the density operator describes the unitary part of the evolution with the free Hamiltonian for an oscillator of angular frequency Ω given by $\hat{H}_0 = \frac{1}{2m}\hat{p}^2 + \frac{1}{2}m\Omega^2\hat{x}^2$. The second term describes the damping with the energy decay rate γ_0. The last term describes the noise introduced due to the coupling to the thermal bath, and gives rise to a diffusion in momentum with a diffusion constant $D_{\mathrm{th}} = \gamma_0\hbar^2(2n_{\mathrm{th}} + 1)/4x_{\mathrm{zp}}^2 \approx \gamma_0 m k_{\mathrm{B}} T$. This term at the same time gives rise to an associated decoherence in position basis. The fact that Eq. S18 gives rise to velocity damping can be seen through evaluation of the expectation values of the position and momentum operators. The expectation value $\langle\hat{O}\rangle \equiv Tr(\hat{\rho}\hat{O})$ for an arbitrary operator $\hat{O}$ under the evolution equation Eq. S18 is given by $\frac{d}{dt}\langle\hat{O}\rangle = \frac{1}{i\hbar}\langle[\hat{O}, \hat{H}_0]\rangle + \frac{1}{i\hbar}\frac{\gamma_0}{2}\langle\{[\hat{O}, \hat{x}], \hat{p}\}\rangle - \frac{D_{\mathrm{th}}}{\hbar^2}\langle[[\hat{O}, \hat{x}], \hat{x}]\rangle$ — the curly brackets represent the anti-commutator. Then, one obtains the equations of motion for the first moments

$$\begin{aligned} \tfrac{d}{dt}\langle\hat{x}\rangle &= \tfrac{1}{m}\langle\hat{p}\rangle \\ \tfrac{d}{dt}\langle\hat{p}\rangle &= -m\Omega^2\langle\hat{x}\rangle - \gamma_0\langle\hat{p}\rangle, \end{aligned} \tag{S19}$$

yielding the dynamical equations for a damped harmonic oscillator $(\frac{d^2}{dt^2} + \gamma_0\frac{d}{dt} + \Omega^2)\langle\hat{x}\rangle = 0$. It can also be seen that the diffusion term enters the equations of motion for the second moment of the momentum: $\frac{d}{dt}\langle\hat{p}^2\rangle \supset 2D_{\mathrm{th}}$.

Projecting Eq. S18 onto position basis and utilizing $\hat{p} = -i\hbar\int dx\,|x\rangle\frac{\partial}{\partial x}\langle x|$, one obtains the evolution equation for the density matrix elements in position basis $\rho \equiv$

$\rho(x, x')$:

$$\begin{aligned}\frac{d}{dt}\rho = &\Big[\frac{1}{i\hbar}\Big(\frac{-\hbar^2}{2m}\big(\frac{\partial^2}{\partial x^2} - \frac{\partial^2}{\partial x'^2}\big) + \frac{m\Omega^2}{2}(x^2 - x'^2)\Big) \\ &- \frac{\gamma_0}{2}(x - x')\big(\frac{\partial}{\partial x} - \frac{\partial}{\partial x'}\big) - \frac{D_{\text{th}}}{\hbar^2}(x - x')^2\Big]\rho.\end{aligned} \tag{S20}$$

In the variables that make the off-diagonal elements explicit, i.e., $\rho \equiv \rho(x + \frac{\Delta}{2}, x - \frac{\Delta}{2})$,

$$\frac{d}{dt}\rho = \Big[\frac{1}{i\hbar}\Big(\frac{-\hbar^2}{m}\frac{\partial^2}{\partial x \partial \Delta} + m\Omega^2 x\Delta\Big) - \gamma_0\Delta\frac{\partial}{\partial \Delta} - \frac{D_{\text{th}}}{\hbar^2}\Delta^2\Big]\rho. \tag{S21}$$

The solution to this equation in steady state (obtained by setting $\frac{d}{dt}\rho = 0$) is of the same form as Eq. S16. The solution is a thermal state with coherence length, position uncertainty and purity given again by Eq. S17.

2.2.2 Quantum feedback damping

Before writing down a master equation for the case of feedback-based damping, we will first address a subtlety associated with the description of the implementation of a velocity feedback force. This arises because of the lack of clarity on how to incorporate the time derivative of position into a Hamiltonian description.

We are interested in implementing a feedback force $F_{\text{fb}}(t) = -m\gamma_{\text{eff}}\frac{d}{dt}x_{\text{obs}}(t)$, containing the time derivative of the continuously observed position $x_{\text{obs}}(t)$ to achieve a velocity damping force with an effective energy damping rate γ_{eff}. Thus we would like to implement the Hamiltonian

$$\begin{aligned}\hat{H} &= \hat{H}_0 + \hat{H}_{\text{fb}} \\ \hat{H}_0 &= \frac{1}{2m}\hat{p}^2 + \frac{1}{2}m\Omega^2 x^2 \\ \hat{H}_{\text{fb}} &= -\hat{x}F_{\text{fb}} = \hat{x}m\gamma_{\text{eff}}\frac{dx_{\text{obs}}}{dt}\end{aligned} \tag{S22}$$

To simplify the description, we first go to an equivalent Hamiltonian formulation of the same physical situation utilizing the unitary transformation $\hat{T} = \exp[i\zeta(\hat{x}, t)/\hbar]$. Now, the states in the new description (primed) are related to the old description by $|\psi'\rangle = \hat{T}|\psi\rangle$, or more generally, the relation to the old density matrix is $\hat{\rho}' = \hat{T}\hat{\rho}\hat{T}^\dagger$. The new Hamiltonian is related to the old one by $\hat{H}' = \hat{T}\hat{H}\hat{T}^\dagger - \frac{\partial\zeta}{\partial t}$, where the nontrivial part of the transformation arises from $\hat{T}\hat{p}\hat{T}^\dagger = \hat{p} - \frac{\partial\zeta}{\partial x}$. A judicious choice of $\zeta(\hat{x}, t)$transforms the problem into what is called a *direct feedback* [10] as opposed to an estimation-based feedback. Choosing $\zeta(\hat{x}, t) = \hat{x}m\gamma_{\text{eff}}x_{\text{obs}}(t)$, we obtain

$$\hat{H}' = \hat{H}_0 - x_{\text{obs}}(t)\gamma_{\text{eff}}\hat{p} \tag{S23}$$

up to an additional operator independent term. The physical observables change form in the new description. While $[\hat{x}, \hat{p}] = i\hbar$ still holds and the role of the position operator remains the same in the new representation, the operator $\hat{p}_{\text{kin}} = \hat{p} - m\gamma_{\text{eff}}x_{\text{obs}}(t)$ becomes the new kinematical momentum that physically corresponds to the observable

$\hat{p}$ in the first description. For example, the physical operator corresponding to the kinetic energy is now $\frac{1}{2m}\hat{p}_{\text{kin}}^2$. The magnitude of the density matrix elements $|\rho(x, x')|$, which is the quantity of interest, remains unchanged between the two representations, since the transformation is only a position-dependent phase shift.

Reformulating the problem in terms of the equivalent Hamiltonian in Eq. S23 significantly simplifies the theoretical analysis. Note that even if we assume white noise for x_{obs} in the frequency range of interest, e.g., due to detection shot noise, the feedback signal has colored noise since x_{obs} is processed through a high-pass filter for differentiation before getting fed back to the system. However, the theoretical description in the new equivalent formalism takes in x_{obs} itself (Eq. S23) — not its derivative as in Eq. S22. This makes a Markovian description possible, i.e. where only white noise needs to be input to the description despite the physical feedback containing colored noise.

Measurement and feedback based on the Hamiltonian in Eq. S23 is well studied [9, 11–14]. Ignoring the negligible intrinsic damping γ_0 with respect to the much larger feedback damping rate γ_{eff} — but keeping the thermal noise due to the intrinsic damping — the unconditional master equation for the density matrix for the current problem can be expressed as

$$\begin{aligned} \frac{d}{dt}\hat{\rho} =& \frac{1}{i\hbar}[H_0, \hat{\rho}] - \frac{1}{i\hbar}\frac{\gamma_{\text{eff}}}{2}[\hat{p}, \hat{x}\hat{\rho} + \hat{\rho}\hat{x}] \\ &- (\frac{D_{\text{th}}}{\hbar^2} + \frac{D_m}{\hbar^2})[\hat{x}, [\hat{x}, \hat{\rho}]] - \frac{D_{\text{fb}}}{\hbar^2}[\hat{p}, [\hat{p}, \hat{\rho}]] \end{aligned} \tag{S24}$$

Unlike the case in Eq. S19, the equations of motion for the expectation values of position and momentum read

$$\begin{aligned} \frac{d}{dt}\langle\hat{x}\rangle &= \frac{1}{m}\langle\hat{p}\rangle - \gamma_{\text{eff}}\langle\hat{x}\rangle, \\ \frac{d}{dt}\langle\hat{p}\rangle &= -m\Omega^2\langle\hat{x}\rangle, \end{aligned} \tag{S25}$$

but, still yield the same dynamical equations for the damped harmonic oscillator $(\frac{d^2}{dt^2} + \gamma_{\text{eff}}\frac{d}{dt} + \Omega^2)\langle\hat{x}\rangle = 0$. In Eq. S24, the second term is responsible for the damping caused by the feedback. The third term now contains an additional diffusion constant D_m that leads to extra momentum diffusion due to the measurement backaction. For our purposes, D_m phenomenologically quantifies the measurement strength as well as the associated positional decoherence strength. The last term leads to a new position diffusion (diffusion constant D_{fb}) due to feeding back a noisy signal to the system — showing up in the equation of motion for the second moment of the position: $\frac{d}{dt}\langle\hat{x}^2\rangle \supset 2D_{\text{fb}}$. For unit detection efficiency, one would have $D_{\text{fb}} = \hbar^2\gamma_{\text{eff}}^2/16D_m$. The master equation for the density matrix elements $\rho \equiv \rho(x + \frac{\Delta}{2}, x - \frac{\Delta}{2})$ now takes the form

$$\begin{aligned} \frac{d}{dt}\rho =& \Big[\frac{1}{i\hbar}\Big(\frac{-\hbar^2}{m}\frac{\partial^2}{\partial x\partial\Delta} + m\Omega^2 x\Delta\Big) - \gamma_{\text{eff}}\big(1 + x\frac{\partial}{\partial x}\big) \\ &- \Big(\frac{D_{\text{th}}}{\hbar^2} + \frac{D_m}{\hbar^2}\Big)\Delta^2 + D_{\text{fb}}\frac{\partial^2}{\partial x^2}\Big]\rho. \end{aligned} \tag{S26}$$

The solution to this equation in steady state is

$$\rho(x+\tfrac{\Delta}{2},x-\tfrac{\Delta}{2}) = \sqrt{\tfrac{1}{2\pi\sigma_x^2}}e^{-x^2/2\sigma_x^2}e^{-\Delta^2/2\sigma_\Delta^2}\ e^{ibx\Delta}. \tag{S27}$$

In the limit in which measurement backaction (radiation pressure noise) is negligible with respect to the thermal noise ($D_m \ll D_{\rm th}$) and in which the imprinted feedback noise is subdominant to thermal noise ($4D_{\rm th}x_{\rm zp}^2/\hbar^2 > D_{\rm fb}/x_{xp}^2$) — as are the cases for our experiment — position uncertainty $\Delta x = \sigma_x$, coherence length $\xi = \frac{1}{2}\sigma_\Delta$ and purity $\mathcal{P} = \frac{\xi}{\Delta x}$ read

$$\begin{aligned} \Delta x &= x_{\rm zp}\left(\tfrac{\gamma_0}{\gamma_{\rm eff}}(2n_{\rm th}+1)+\tfrac{\Delta x_{\rm fb}^2}{x_{\rm zp}^2}\right)^{1/2} \equiv x_{\rm zp}\sqrt{2n+1}, \\ \xi &= x_{\rm zp}\left((2n+1)+\tfrac{\Delta x_{\rm fb}^2}{x_{\rm zp}^2}Q_{\rm eff}^{-2}\right)^{-1/2} = s\ x_{\rm zp}/\sqrt{2n+1}, \\ \mathcal{P} &= s/(2n+1). \end{aligned} \tag{S28}$$

Here, the quality factor is defined as $Q_{\rm eff} = \Omega/\gamma_{\rm eff}$, the measurement noise imprinted by the feedback as $\Delta x_{\rm fb} = (D_{\rm fb}/\gamma_{\rm eff})^{1/2}$ and the correction factor due to the imprinted measurement noise as $s = (1+Q_{\rm eff}^{-2}\frac{\Delta x_{\rm fb}^2}{\Delta x^2})^{-1/2}$. The definition for calculating the effective mean thermal excitation quanta n is also indicated. We see that the feedback itself acts to reduce the initial excitation numbers by a factor of $\frac{\gamma_0}{\gamma_{\rm eff}}$. On the other hand, looking at the first of Eqs. S28, we see that the imprinted measurement noise $\Delta x_{\rm fb}$ due to the feedback tends to do the opposite action — but it is subdominant in our range of parameters. The second of Eqs. S28 shows that the negative effect of the imprinted measurement noise starts to get amplified from the perspective of the coherence length as one approaches critical damping. Depending on the magnitude of the feedback noise, this amplified contribution could thus become important. Nevertheless in our range of parameters we still have $s \approx 1$; see main text. Eq. S28 is to be contrasted with the thermal bath damping results, Eq. S17, where the excess terms due to feedback are absent.

2.2.3 Experimental extraction of coherence length and purity

First, note that the utilized master equation description assumes an infinite feedback bandwidth. Given that our pendulum operates at 18 Hz, and our feedback loop maintains the velocity damping character up to 1.5 kHz, the infinite bandwidth assumption holds well, as the susceptibility of our torsional oscillator rapidly declines above resonance. In addition, note that excess detection noise or excess vibration noise can be bundled into the phenomenological momentum and position diffusion coefficients $D_{\rm th}$ and $D_{\rm fb}$ without the need for a change in the model.

To translate the results of the linear harmonic oscillator into those of a torsional oscillator, we simply need to replace the linear zero-point fluctuation level $x_{\rm zp}$ with its angular counterpart $\theta_{\rm zp} = \sqrt{\hbar/2I\Omega}$, where I is the moment of inertia. Then, we

obtain the angular analogues of Eqs. S28:

$$\begin{aligned}
\Delta\theta &= \theta_{zp}\left(\tfrac{\gamma_0}{\gamma_{eff}}(2n_{th}+1)+\tfrac{\Delta\theta_{fb}^2}{\theta_{zp}^2}\right)^{1/2} \equiv \theta_{zp}\sqrt{2n+1},\\
\xi_\theta &= s\,\theta_{zp}/\sqrt{2n+1},\\
\mathcal{P} &= s/(2n+1)
\end{aligned} \tag{S29}$$

in terms of the effective mean excitation quanta n and the additional suppression factor $s = \left(1+Q_{eff}^{-2}\ \Delta\theta_{fb}^2/\Delta\theta^2\right)^{-1/2}$. This suppression factor has an effect only when the oscillator becomes near-critically damped, with the effect increasing for overdamping. The variance ratio $\Delta\theta_{fb}^2/\Delta\theta^2$ is experimentally available through the imprinted noise modeling in the context of Fig. 2(b) that utilizes Eq. S10. Following the method discussed in the main text for extracting ratios of variances through their PSDs (Eq. 4), this ratio can be evaluated as

$$\frac{\Delta\theta_{fb}^2}{\Delta\theta^2} = \frac{\int_{f_1}^{f_2} |\chi_{imp}(f)|^2 S_{\theta\theta}^{imp}(f)\ df}{\int_{f_1}^{f_2} S_{\theta\theta}(f)\ df}, \tag{S30}$$

again with $f_1 = 8$ Hz and $f_2 = 28$ Hz. Recall that $\chi_{imp}(f)$ is the transfer function in Eq. S9 for the experimentally characterized measurement imprecision noise $S_{\theta\theta}^{imp}(f)$.

Eq. S29 is what we use to extract coherence angles ξ_θ and purity $\mathcal{P}$ in the main text. The conversion of this angular coherence to a linear coherence length ξ for the tip of the pendulum bar is accomplished by multiplying by half the length of the pendulum bar.

2.3 Figure of merit η for different systems

Detailed comparisons of systems with vastly differing properties for their utility at the quantum-gravity interface can be tedious. Especially, when the aspect ratios of the objects become large, a calculation is needed to assess the distributed nature of the interaction for proper comparison. Nevertheless, it is still possible to draw general conclusions by classifying objects into a few basic geometrical categories. A further complication arises when considering the oscillation-mode structures, e.g., higher-order standing-wave modes in a membrane, or standing-wave modes in a cantilever. In order to prevent underestimations of η values, we make a gravitationally-overestimating assumption, and pretend that the entire object moves uniformly with the amplitude of the relevant mode, dispensing of mode structures for ease of comparison.

2.3.1 On the adapted form of η

In references [15] and [16], the authors theoretically examine the onset of entanglement between two gravitationally interacting oscillators using logarithmic negativity to quantify the effect. In these works, oscillators are subjected to continuous measurement-based state purification in presence of thermal noise, with additional inclusion of measurement-based feedback damping in Ref. [16]. The authors reach

identical conclusions for the regime of physical parameters required for observing entanglement in their systems — within a factor of $\sqrt{2}$ specific to the optomechanical systems considered. The authors of [15] further show that this parameter regime is equally applicable to free particles (instead of oscillators), pointing to the universality of the conditions. For Ref. [15], the condition is given in their appendix Eq. A14, whereas for Ref. [16] this condition is given in the sentence following their Eq. 5; namely, their statement "$\Omega\epsilon > 4\sqrt{2}\gamma_m n_{\rm th}^{+}$". Our figure-of-merit η in Eq. 1 and its form in terms of the ground-state-entanglement-rate precisely replicate these equations once the definition of quantum coherence length or decoherence rate, respectively, are introduced.

It is instructive to see how η relates to decoherence rate and how feedback damping enters Eq. 1. Note that, in this equation, γ refers to the total damping rate, i.e., to our $\gamma_{\rm eff}$ that includes feedback damping, and $\xi^2 = \frac{x_{\rm zp}^2}{2n+1}$ refers to the (squared) coherence length under feedback control, where $n = n_{\rm th}\frac{\gamma_0}{\gamma_{\rm eff}}$ is the mean excitation number under feedback, and γ_0 is the bare damping rate in absence of feedback. Therefore η can be written in the two following alternate forms:

$$\eta^2 = \frac{\xi^2|\nabla F|}{\hbar\gamma_{\rm eff}} = \frac{\Gamma_{\rm ent}^{(0)}}{2\Gamma_{\rm dec}}. \tag{S31}$$

Here, $\Gamma_{\rm ent}^{(0)} = x_{\rm zp}^2|\nabla F|/\hbar$ is the entanglement rate referred to in the main text when the oscillators are in their ground states, and $\Gamma_{\rm dec} = n_{\rm th}\gamma_0$ is the decoherence rate, assumed to be limited by thermal noise—additional rates due to other decoherence sources can be added if desired. The second form of Eq. S31 makes it manifest that, fundamentally, feedback damping ($\gamma_{\rm eff}$) does not enter the value of η since neither the numerator nor the denominator depends on it. However the first form of the equation makes much more connection with experiments, as the requirement for actually generating entanglement is the ability to control and maintain the systems in near-pure quantum states [16]. The first expression is written in terms of post-control measurable quantities, whereas the second form is written in terms of quantities that do not specify anything about achieved level of control, but can be extracted from free-running properties of the oscillator. Therefore we utilize the first expression that involves achieved coherence lengths for experimental benchmarking.

2.3.2 Geometry factors for evaluating $|\nabla F|$

The quantity of interest that enters η is the force gradient between the two objects with respect to their separation d. Given two extended objects and their gravitational interaction energy $U[d]$ that appears in the system Hamiltonian, the force gradient between the objects is the second derivative of the interaction energy: $|\nabla F| = |\partial^2 U/\partial d^2|$. Taking two identical objects with uniform mass densities and equal masses m, we will calculate the force gradients for three umbrella cases to cover all types of objects. The 3D calculation is intended to cover spherical, and other small aspect ratio objects; the 2D calculation is intended to cover membrane-like objects; and the 1D calculation is intended to cover objects like beams, rods, and cantilevers. Comparing the geometry functions obtained in each case will show us

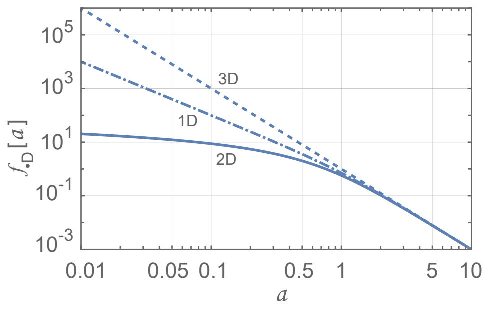

Fig. S5 Comparison of the geometry factors $f_{\cdot\mathrm{D}}[a]$ that describe the force gradient $|\nabla F|$ for different object geometries. Saturation behavior of the force gradient is illustrated as objects approach each other (dimensionless a decreasing). 1D: line-like objects, 2D: plate-like objects, 3D: point-like objects.

how the associated force gradients saturate as the object separation approaches the major physical dimensions of each type of object.

3D – Two spherical point particles of mass m each, separated by d.

The gravitational interaction energy of two point particles can be written as $U[d] = -\frac{Gm^2}{d}$. The resulting force gradient is $|\nabla F| = 2\frac{Gm^2}{d^3} = 2\frac{Gm^2}{L^3} f_{3\mathrm{D}}[d/L]$. The factor

$$f_{3\mathrm{D}}[a] = 1/a^3 \tag{S32}$$

encodes the geometry of the problem, and is introduced through the arbitrary length scale L to compare with the other cases, where real length scales do exist.

2D – Two parallel thin disks with diameter L and mass m each, separated axially by d.

The problem of charged interacting disks in electrostatics has been well studied [17]. Borrowing from the results of these works, the analogous gravitational interaction energy of two disks can be written as $U[d, R] = -\frac{Gm^2}{L} g_{2\mathrm{D}}[d/L]$, where the function $g_{2\mathrm{D}}$ encoding the geometry of the problem is given in terms of the Bessel function $J_1[u]$ of the first kind as $g_{2\mathrm{D}}[a] = 8\int_0^\infty du \left(\frac{J_1[u]}{u}\right)^2 e^{-2au}$. The resulting force gradient is $|\nabla F| = 2\frac{Gm^2}{L^3} f_{2\mathrm{D}}[d/L]$, where the geometry factor is

$$f_{2\mathrm{D}}[a] = 16\int_0^\infty du\ J_1[u]^2 e^{-2au}. \tag{S33}$$

The integral transform in Eq. S33 can be written in terms of the Legendre function of the second kind $Q^0_{\nu-1/2}[x]$, with $\nu = 1$ [18]. Dropping the upper index to simplify the notation, this yields $f_{2\mathrm{D}}[a] = \frac{16}{\pi} Q_{1/2}[2a^2+1]$. Utilizing tabulated asymptotic expressions $Q_\nu\left[x \to \infty\right] = \frac{\sqrt{\pi}\Gamma(\nu+1)}{\Gamma(\nu+3/2)(2x)^{\nu+1}}$ and $Q_\nu[x \to 1+] = \frac{1}{2}\ln 2 - \gamma_\mathrm{E} - \psi\left(\nu+1\right) -$

$\frac{1}{2}\ln(x-1)$, where γ_{E} is Euler's constant and ψ is the Digamma function, we reach

$$f_{2\mathrm{D}}[a \to \infty] = \frac{1}{a^3}. \tag{S34}$$

$$f_{2\mathrm{D}}[a \to 0] \approx \frac{16}{\pi}\left(-0.61 - \ln[a]\right). \tag{S35}$$

1D – Two parallel thin rods with length L and mass m each, separated by d.

The problem of charged interacting rods in electrostatics has been well studied [19]. Borrowing from the results of these works, the analogous gravitational interaction energy of two rods can be written as $U[d,L] = -\frac{Gm^2}{L} g_{1\mathrm{D}}[d/L]$, where the function $g_{1\mathrm{D}}[a] = ln[\frac{1+\sqrt{1+a^2}}{-1+\sqrt{1+a^2}}] - 2\sqrt{1+a^2} + 2a$ encodes the geometry of the problem. The resulting force gradient is $|\nabla F| = 2\frac{Gm^2}{L^3} f_{1\mathrm{D}}[d/L]$, where the geometry factor is

$$f_{1\mathrm{D}}[a] = \frac{1}{a^2\sqrt{1+a^2}}. \tag{S36}$$

Given that the prefactors of $|\nabla F|$ have been arranged in the same form with respect to the reference length in all cases, to study the saturation behavior of the force gradient, we simply need to compare the functions $f_{\cdot\mathrm{D}}[a]$; see Figure S5. For large separations, objects behave as if they are point-like particles (3D), but once the separation approaches the object's major dimension, force gradients begin saturation. There is no saturation for the 3D case. The saturation is complete in the case of membranes (2D) — there is virtually no benefit in bringing two parallel membranes closer to each other than their major dimension. For the case of rods (1D), the improvement with respect to separation drastically decreases at the saturation point, nevertheless still showing some improvement.

Thus, the best strategy for an accurate comparison is to utilize the suitable geometry factor to calculate the force gradient at the separation point that the two objects touch — assuming that this dimension is larger than 50 μm. As discussed in the main text, the closest approach of two objects will be limited to 50 μm, as screening electromagnetic interactions to make gravity dominant below that level will not be meaningful.

2.3.3 Parameters used for η comparisons

In Table S1, we tabulate the numbers utilized in the comparison for the η parameters for the different oscillators shown in Fig. 3. These are the numbers that go directly into Equation 1. Recall the geometric quantities used to calculate the force gradient $|\nabla F|$: the center separation between objects d and the major dimension of the object L — e.g., diameter of a sphere, side length of a square membrane, or length of a cantilever.

Osc.	m (kg)	f (Hz)	$\gamma/2\pi$ (Hz)	n	$\mathcal{P}$	$x_{\mathrm{zp}}(m)$	ξ (m)	$^{\dagger}L$ (m)	$^{*}d$ (m)	$\lvert\nabla F\rvert$ (N m^{-1})	η
a)	1.2×10^{-18}	5.7×10^{4}	7×10^{3}	0.68	0.42	1.1×10^{-11}	2.5×10^{-11}	$^{3D}10^{-7}$	5×10^{-5}	1.5×10^{-33}	4.6×10^{-13}
b)	2.8×10^{-18}	3.1×10^{5}	4.8×10^{4}	0.43	0.54	3.1×10^{-12}	2.3×10^{-12}	$^{3D}1.4\times10^{-7}$	5×10^{-5}	8.5×10^{-33}	3.6×10^{-14}
c)	$^{\ddagger}2.9\times10^{-15}$	4.3×10^{6}	5.2×10^{4}	5.3	8.6×10^{-2}	2.6×10^{-14}	7.6×10^{-15}	$^{1D}6.5\times10^{-5}$	5×10^{-5}	5.5×10^{-27}	9.6×10^{-14}
d)	9.4×10^{-13}	63	6×10^{4}	1.6×10^{4}	3.1×10^{-5}	3.8×10^{-13}	2.1×10^{-15}	$^{3D}10^{-5}$	5×10^{-5}	9.4×10^{-22}	10^{-11}
e)	$^{\ddagger}2.3\times10^{-12}$	1.1×10^{6}	2×10^{3}	0.29	0.63	1.8×10^{-15}	1.4×10^{-15}	$^{2D}7\times10^{-4}$	5×10^{-7}	2.1×10^{-23}	5.7×10^{-12}
f)	$^{\ddagger}14\times10^{-12}$	2.7×10^{5}	80	14	3.4×10^{-2}	1.5×10^{-15}	2.7×10^{-16}	$^{1D}9\times10^{-5}$	5×10^{-5}	1.0×10^{-19}	3.8×10^{-10}
g)	$^{\ddagger}4.3\times10^{-11}$	7.1×10^{5}	4.9×10^{3}	0.84	0.37	5.3×10^{-16}	3.2×10^{-16}	$^{2D}10^{-3}$	5×10^{-5}	3.0×10^{-21}	9.8×10^{-12}
h)	5.6×10^{-9}	212	8.2×10^{-6}	2.5×10^{8}	2.0×10^{-9}	2.7×10^{-15}	$^{1}1.2\times10^{-19}$	$^{3D}10^{-4}$	10^{-4}	4.2×10^{-15}	1.1×10^{-7}
i)	$^{\ddagger}1.6\times10^{-8}$	5.9×10^{9}	8.4×10^{3}	$^{2}2.6$	0.75	3.0×10^{-19}	$^{2}2.1\times10^{-18}$	$^{1D}4\times10^{-4}$	5×10^{-5}	3.4×10^{-14}	1.6×10^{-10}
This	10^{-6}	18	31	2.8×10^{5}	1.4×10^{-6}	$^{3}1.2\times10^{-15}$	$^{3}1.2\times10^{-18}$	$^{1D}2\times10^{-3}$	5×10^{-4}	2.6×10^{-13}	4.3×10^{-9}
level 1				1.1×10^{2}	3.5×10^{-3}		6.0×10^{-17}				2.1×10^{-7}
level 2		2×10^{2}		1	0.58	2.0×10^{-16}	3.6×10^{-16}				1.3×10^{-6}
level 3			3.1×10^{-3}								1.3×10^{-4}
j)	5.0×10^{-6}	6.6×10^{2}	2.4×10^{2}	4.7×10^{5}	1.1×10^{-6}	5.0×10^{-17}	5.2×10^{-20}	$^{2D}3.0\times10^{-3}$	$^{4}5\times10^{-4}$	7.8×10^{-13}	1.1×10^{-10}
k)	1.0×10^{-3}	10^{3}	9.3×10^{3}	1.4×10^{5}	3.6×10^{-6}	2.9×10^{-18}	5.5×10^{-21}	$^{2D}1.2\times10^{-4}$	$^{5}4.0\times10^{-3}$	2.6×10^{-10}	1.1×10^{-10}
l)	$^{\ddagger}10$	1.5×10^{2}	1.5×10^{2}	11	4.4×10^{-2}	7.5×10^{-20}	1.6×10^{-20}	$^{3D}0.35$	0.35	$3.1\times10{-7}$	2.8×10^{-8}
m)	$^{\ddagger}1.1\times10^{3}$	9.1×10^{2}	21	3.9×10^{3}	1.3×10^{-4}	2.9×10^{-21}	3.3×10^{-23}	$^{1D}3.0$	0.4	3.3×10^{-4}	5.1×10^{-9}

Table S1 Numbers utilized for different oscillators for η comparison in Fig. 3. Experiments: (a) nanosphere [20], (b) nanosphere [21], (c) nanobeam [22], (d) microsphere [23], (e) membrane [24], (f) cantilever [25], (g) membrane [26], (h) superconducting microsphere [27], (i) acoustic resonator [28], (j) pendulum [29], (k) pendulum [30], (l) LIGO pendulums [31], (m) bar resonator [32]. Parameters for 'This' work and the assumed future numbers under 'level 1', 'level 2' and 'level 3' are also indicated.

† Object's major characteristic dimension.
* Smaller of the two: center separation when objects touch, or 50 μm due to shielding arguments.
1D Utilized geometry factor is f_{1D}.
2D Utilized geometry factor is f_{2D}.
3D Utilized geometry factor is f_{3D}.
‡ These are effective masses provided by the respective references.
1 Based on lowest observed motional temperature of 2.5 K; private communication.
2 Coherence length calculated based on Schrodinger cat separation $\bar{n} = 2.6$ with $\xi = 4x_{\mathrm{zp}}\sqrt{\bar{n}+1/2}$.
3 $x_{\mathrm{zp}} \equiv \theta_{\mathrm{zp}} l$ and $\xi \equiv \xi_{\theta} l$ are calculated based on pendulum lever arm of $l = 1$ mm.
4 For pendulum dimensions refer to ref. [33].
5 Pendulum dimensions extracted from ref. [34].

2.3.4 On the potential future performance

The main parameters that go into calculating the future performances for the three levels indicated in Fig. 3 are summarized in Table S1. Here, we discuss the general requirements for ground state cooling and provide additional information on the utilized parameters.

The requirements for feedback-control-based ground state cooling has been discussed in many works including refs. [35–38]. Particularly relevant for the case with structural damping are the works [37, 38]. In summary, one needs to be quantum radiation pressure noise (QRPN) limited while being able to sense the motion at the optical shot noise limit, and have a sufficiently large apparent quality factor to suppress the motion, or said differently, satisfy the $Q \cdot f > k_{\mathrm{B}}T/h$ criterion [37]. We recall for the reader the special role played by structural damping discussed in the main text as per Eq. 3: Upon frequency shifting, the apparent quality factor becomes $Q_{\mathrm{app}} = (\omega_{\mathrm{eff}}/\omega_0)^2 \, Q_0$, with one of the factors of $\omega_{\mathrm{eff}}/\omega_0$ originating from the reduction in structural damping $\gamma_{\mathrm{app}} = (\omega_{\mathrm{eff}}/\omega_0)\gamma_0$, and the other one simply from the definition $Q_{\mathrm{app}} = \omega_{\mathrm{eff}}/\gamma_{\mathrm{app}}$. Further, notice that as the frequency is shifted up, the initial thermal occupation number $k_{\mathrm{B}}T/\hbar\omega_{\mathrm{eff}}$ becomes lower to begin with. The combination of the apparent quality factor increase and the reduction of initial excitations with frequency shifting translates into the requirement $Q_{\mathrm{app}}\omega_{\mathrm{eff}} = (\omega_{\mathrm{eff}}/\omega_0)^3 Q_0\omega_0 > k_{\mathrm{B}}T/\hbar$, where $k_{\mathrm{B}}T/\hbar \sim 4\times10^{13}$ s^{-1} at room temperature. Note that another statement usually associated with ground state cooling capability is the requirement of a measurement sensitivity that is a factor of n_{th} below that of the zero-point motion. This statement is covered by the requirements we already laid out [37]. With these preliminaries, we now discuss and see how one can bridge the current $n \sim 10^5$ to $n \sim 1$ excitations.

Each step assumes structural thermal noise limited operation, requiring the mitigation of technical disturbances such as seismic vibrations at relevant levels. Noise due to background gas damping is already eliminated at any level with the current vacuum performance: At ambient pressures, we observe a torsional Q factor nearing 10, indicating a damping rate of $\sim 2\pi \times 1$ Hz at 1 bar; hence at 10^{-9} mbar, the background gas collision limit is $\gamma_{\mathrm{app}} \sim 2\pi \times 1$ pHz—better than the values to be assumed below.

Level 1: Use a 1-μm-diameter suspension fiber, operating near $Q_0 = 2 \times 10^4$— a readily demonstrated technology [39]. This realizes a 65 mHz torsional oscillator with $\gamma_0 = 2\pi \times 3\mu$Hz. Shift the frequency to 18 Hz via feedback, obtaining $Q_{\mathrm{app}} = 1.5 \times 10^9$, $\gamma_{\mathrm{app}} = 2\pi \times 10$ nHz, and $Q_{\mathrm{app}}\omega_{\mathrm{eff}} = 1.7 \times 10^{11}$ s^{-1}. This also requires increasing the optical lever detection power to 10-mW for the shot noise in the lever to be below the thermal noise level of the torsional motion at 18 Hz, such that no appreciable excess detection noise is imprinted through the feedback loop. Being shot noise limited in the lever of course requires improving the technical pointing noise in the lever by about 2 orders of magnitude, which can for example be done via feedback stabilizing the pointing out of the fiber launcher. At critical feedback damping, γ_{eff} remains at the same value as in this work, but the oscillator is cooled 3000-fold further to $n = 100$, becoming more pure. This also manifests itself in a 3000-fold increase in ξ^2, resulting in a 55-fold increase in η.

Level 2: Going to a regime where QRPN dominates over the thermal noise while the measurements take place at shot-noise level will be quite challenging with an optical

lever. Optical cavities are much more effective for this task as the required input powers go down with the square of cavity finesse. We refer the reader to ref. [40] for how torsional motion can be coupled to an optical cavity and QRPN domination could be achieved with microwatts of input optical power. With such a system, optical spring implementations can also become more effective, with operations at kilohertz levels already demonstrated [29, 30]. Thus, in a cavity, shifting the mechanical frequency to 200 Hz via an optical spring can result into $Q_{\mathrm{app}} = 2 \times 10^{11}$ and $Q_{\mathrm{app}}\omega_{\mathrm{eff}} = 2.4 \times 10^{14}\ \mathrm{s}^{-1}$, satisfying the requirements for ground state cooling in a room temperature environment. Coherence length now approaches the zero-point fluctuations associated with a 200 Hz oscillator and the purity approaches 1. Further, if cooling to $n = 1$ is assumed, then keeping the same absolute feedback damping rate suffices, operating at $Q_{\mathrm{eff}} = 6$, resulting in a roughly 2-fold increase in η.

Level 3: Operate inside a dilution refrigerator at 30 mK. This reduces the initial thermal occupation number by a factor of 10^4, in turn relaxing the required feedback damping rate by the same amount, and boosting η up a 100-fold. Operation in a cryogenic environment will likely come with many new technical challenges, but fortunately many examples exist (see Fig. 3.). The low heat loads in the fridge due to low light level operation, and the relaxed damping requirements can be considered encouraging.

Supplementary

References

[1] Treps, N., Grosse, N., Bowen, W.P., Fabre, C., Bachor, H.-A., Lam, P.K.: A quantum laser pointer. Science **301**(5635), 940–943 (2003)

[2] Fuchs, T.M., Uitenbroek, D.G., Plugge, J., Halteren, N., Soest, J.-P., Vinante, A., Ulbricht, H., Oosterkamp, T.H.: Measuring gravity with milligram levitated masses. Science Advances **10**(8), 2949 (2024)

[3] Komori, K., Enomoto, Y., Ooi, C.P., Miyazaki, Y., Matsumoto, N., Sudhir, V., Michimura, Y., Ando, M.: Attonewton-meter torque sensing with a macroscopic optomechanical torsion pendulum. Phys. Rev. A **101**, 011802 (2020) https://doi.org/10.1103/PhysRevA.101.011802

[4] Huyet, G., Franke-Arnold, S., Barnett, S.M.: Superposition states at finite temperature. Physical Review A **63**(4), 043812 (2001)

[5] Franke-Arnold, S., Huyet, G., Barnett, S.M.: Measures of coherence for trapped matter waves. Journal of Physics B: Atomic, Molecular and Optical Physics **34**(5), 945 (2001)

[6] Agarwal, G.: Master equations in phase-space formulation of quantum optics. Physical Review **178**(5), 2025 (1969)

[7] Savage, C., Walls, D.: Damping of quantum coherence: the master-equation approach. Physical Review A **32**(4), 2316 (1985)

[8] Savage, C., Walls, D.: Quantum coherence and interference of damped free particles. Physical Review A **32**(6), 3487 (1985)

[9] Wiseman, H.M., Milburn, G.J.: Quantum Measurement and Control. Cambridge university press, Cambridge UK (2009)

[10] Doherty, A.C., Jacobs, K.: Feedback control of quantum systems using continuous state estimation. Physical Review A **60**(4), 2700 (1999)

[11] Caves, C.M., Milburn, G.J.: Quantum-mechanical model for continuous position measurements. Physical Review A **36**(12), 5543 (1987)

[12] Wiseman, H.M.: Quantum theory of continuous feedback. Physical Review A **49**(3), 2133 (1994)

[13] Hopkins, A., Jacobs, K., Habib, S., Schwab, K.: Feedback cooling of a nanomechanical resonator. Physical Review B **68**(23), 235328 (2003)

[14] Rouillard, A., Reddy, A., Bassa, H., Maharaj, S., Diosi, L., Konrad, T.:

Measurement-based feedback control of a quantum system in a harmonic potential. arXiv preprint arXiv:2212.12292 (2022)

[15] Miao, H., Martynov, D., Yang, H., Datta, A.: Quantum correlations of light mediated by gravity. Phys. Rev. A **101**, 063804 (2020) https://doi.org/10.1103/PhysRevA.101.063804

[16] Miki, D., Matsumura, A., Yamamoto, K.: Feasible generation of gravity-induced entanglement by using optomechanical systems. Physical Review D **110**(2), 024057 (2024)

[17] Ciftja, O.: Electrostatic interaction energy between two coaxial parallel uniformly charged disks. Results in Physics **15**, 102684 (2019)

[18] *NIST Digital Library of Mathematical Functions. https://dlmf.nist.gov/, Release 1.2.4 of 2025-03-15. F. W. J. Olver, A. B. Olde Daalhuis, D. W. Lozier, B. I. Schneider, R. F. Boisvert, C. W. Clark, B. R. Miller, B. V. Saunders, H. S. Cohl, and M. A. McClain, eds. https://dlmf.nist.gov/*

[19] Ciftja, O., Paredes, G., Griffin, M.: Mathematical expressions for a system of two identical uniformly charged rods. Physica Scripta **89**(11), 115803 (2014)

[20] Rossi, M., Militaru, A., Carlon Zambon, N., Riera-Campeny, A., Romero-Isart, O., Frimmer, M., Novotny, L.: Quantum delocalization of a levitated nanoparticle. Phys. Rev. Lett. **135**, 083601 (2025) https://doi.org/10.1103/2yzc-fsm3

[21] Delić, U., Reisenbauer, M., Dare, K., Grass, D., Vuletić, V., Kiesel, N., Aspelmeyer, M.: Cooling of a levitated nanoparticle to the motional quantum ground state. Science **367**(6480), 892–895 (2020) https://doi.org/10.1126/science.aba3993

[22] Wilson, D.J., Sudhir, V., Piro, N., Schilling, R., Ghadimi, A., Kippenberg, T.J.: Measurement-based control of a mechanical oscillator at its thermal decoherence rate. Nature **524**(7565), 325–329 (2015) https://doi.org/10.1038/nature14672

[23] Monteiro, F., Li, W., Afek, G., Li, C.-l., Mossman, M., Moore, D.C.: Force and acceleration sensing with optically levitated nanogram masses at microkelvin temperatures. Physical Review A **101**(5), 053835 (2020)

[24] Rossi, M., Mason, D., Chen, J., Tsaturyan, Y., Schliesser, A.: Measurement-based quantum control of mechanical motion. Nature **563**(7729), 53–58 (2018) https://doi.org/10.1038/s41586-018-0643-8

[25] Zoepfl, D., Juan, M.L., Diaz-Naufal, N., Schneider, C.M.F., Deeg, L.F., Sharafiev, A., Metelmann, A., Kirchmair, G.: Kerr enhanced backaction cooling in magnetomechanics. Phys. Rev. Lett. **130**, 033601 (2023) https://doi.org/10.1103/PhysRevLett.130.033601

[26] Underwood, M., Mason, D., Lee, D., Xu, H., Jiang, L., Shkarin, A.B., Børkje, K., Girvin, S.M., Harris, J.G.E.: Measurement of the motional sidebands of a nanogram-scale oscillator in the quantum regime. Phys. Rev. A **92**, 061801 (2015) https://doi.org/10.1103/PhysRevA.92.061801

[27] Hofer, J., Gross, R., Higgins, G., Huebl, H., Kieler, O.F., Kleiner, R., Koelle, D., Schmidt, P., Slater, J.A., Trupke, M., Uhl, K., Weimann, T., Wieczorek, W., Aspelmeyer, M.: High-q magnetic levitation and control of superconducting microspheres at millikelvin temperatures. Phys. Rev. Lett. **131**, 043603 (2023) https://doi.org/10.1103/PhysRevLett.131.043603

[28] Bild, M., Fadel, M., Yang, Y., Von Lüpke, U., Martin, P., Bruno, A., Chu, Y.: Schrödinger cat states of a 16-microgram mechanical oscillator. Science **380**(6642), 274–278 (2023) https://doi.org/10.1126/science.adf7553

[29] Matsumoto, N., Komori, K., Ito, S., Michimura, Y., Aso, Y.: Direct measurement of optical-trap-induced decoherence. Phys. Rev. A **94**, 033822 (2016) https://doi.org/10.1103/PhysRevA.94.033822

[30] Corbitt, T., Wipf, C., Bodiya, T., Ottaway, D., Sigg, D., Smith, N., Whitcomb, S., Mavalvala, N.: Optical dilution and feedback cooling of a gram-scale oscillator to 6.9 mk. Phys. Rev. Lett. **99**, 160801 (2007) https://doi.org/10.1103/PhysRevLett.99.160801

[31] Whittle, C., *et al.*: Approaching the motional ground state of a 10-kg object. Science **372**(6548), 1333–1336 (2021) https://doi.org/10.1126/science.abh2634

[32] Vinante, A., Bignotto, M., Bonaldi, M., Cerdonio, M., Conti, L., Falferi, P., Liguori, N., Longo, S., Mezzena, R., Ortolan, A., Prodi, G.A., Salemi, F., Taffarello, L., Vedovato, G., Vitale, S., Zendri, J.-P.: Feedback cooling of the normal modes of a massive electromechanical system to submillikelvin temperature. Phys. Rev. Lett. **101**, 033601 (2008) https://doi.org/10.1103/PhysRevLett.101.033601

[33] Matsumoto, N., Cataño-Lopez, S.B., Sugawara, M., Suzuki, S., Abe, N., Komori, K., Michimura, Y., Aso, Y., Edamatsu, K.: Demonstration of displacement sensing of a mg-scale pendulum for mm- and mg-scale gravity measurements. Phys. Rev. Lett. **122**, 071101 (2019) https://doi.org/10.1103/PhysRevLett.122.071101

[34] Corbitt, T.R.: Quantum noise and radiation pressure effects in high power optical interferometers. PhD thesis, Massachusetts Institute of Technology (2008)

[35] Shin, D.-C., Hayward, T.M., Fife, D., Menon, R., Sudhir, V.: Active laser cooling of a centimeter-scale torsional oscillator. Optica **12**(4), 473–478 (2025) https://doi.org/10.1364/OPTICA.548098

[36] Pluchar, C.M., Agrawal, A.R., Wilson, D.J.: Quantum-limited optical lever measurement of a torsion oscillator. Optica **12**(3), 418–423 (2025) https://doi.org/

10.1364/OPTICA.549814

[37] Michimura, Y., Komori, K.: Quantum sensing with milligram scale optomechanical systems. The European Physical Journal D **74**(6), 126 (2020) https://doi.org/10.1140/epjd/e2020-10185-5

[38] Komori, K., Ďurovčíková, D., Sudhir, V.: Quantum theory of feedback cooling of an anelastic macromechanical oscillator. Phys. Rev. A **105**, 043520 (2022) https://doi.org/10.1103/PhysRevA.105.043520

[39] Cataño-Lopez, S.B., Santiago-Condori, J.G., Edamatsu, K., Matsumoto, N.: High-q milligram-scale monolithic pendulum for quantum-limited gravity measurements. Phys. Rev. Lett. **124**, 221102 (2020) https://doi.org/10.1103/PhysRevLett.124.221102

[40] Agafonova, S., Mishra, U., Diorico, F., Hosten, O.: Zigzag optical cavity for sensing and controlling torsional motion. Phys. Rev. Res. **6**, 013141 (2024) https://doi.org/10.1103/PhysRevResearch.6.013141
